# The Role of Protein Intake on the Total Milk Protein in Lead-Exposed Lactating Mothers

**DOI:** 10.3390/nu15112584

**Published:** 2023-05-31

**Authors:** Linda Ratna Wati, Djanggan Sargowo, Tatit Nurseta, Lilik Zuhriyah

**Affiliations:** 1 Doctoral Program in Medical Science, Faculty of Medicine, Universitas Brawijaya, Malang 65145, East Java, Indonesia; 2 Department of Midwifery, Faculty of Medicine, Universitas Brawijaya, Malang 65145, East Java, Indonesia; 3 Department of Cardiology, Faculty of Medicine, Universitas Brawijaya, Universitas Brawijaya Hospital, Malang 65145, East Java, Indonesia; 4 Department of Obstetrics and Gynecology, Faculty of Medicine, Universitas Brawijaya, Malang 65145, East Java, Indonesia; 5 Department of Public Health, Faculty of Medicine, Universitas Brawijaya, Malang 65145, East Java, Indonesia

**Keywords:** breast milk, lead exposure, milk protein, protein intake

## Abstract

Protein is an essential macronutrient for the growth and development of infants. Protein levels in lactating mothers are dynamic and influenced by various factors, particularly the environment and maternal characteristics. Therefore, this study aimed to evaluate the complex correlation between maternal blood lead levels (BLLs), maternal diet, and total milk protein. The Kruskal–Wallis test was used to compare total milk protein in the three groups of lead exposure, while Spearman’s correlation was used to assess the correlation between maternal diet, BLLs, and total milk protein. The multivariate analysis used multiple linear regression. The results showed that the median of maternal BLLs and total milk protein were 3.3 µg/dL and 1.07 g/dL, respectively. Maternal protein intake and current BMI had a positive correlation with total milk protein, while BLLs had a negative correlation. BLLs ≥ 5 μg/dL had the most significant impact on reducing the total milk protein (*p* = 0.032). However, increasing maternal protein intake can effectively maintain total milk protein levels in mothers with BLLs under 5 μg/dL (*p* < 0.001). It is crucial to measure BLLs in lactating mothers residing in areas exposed to lead because high maternal protein intake can only maintain total milk protein levels when the BLLs are <5 μg/dL.

## 1. Introduction

Breast milk (BM) is an excellent source of nutrients to support the growth and development of infants in their early stage of life, due to its abundance of essential compounds [1]. It contains various significant components such as lipids, proteins, and carbohydrates [2]. Mature breast milk is comprised of approximately 6.7 to 7.8 g/dL of carbohydrates, 3.2 to 3.6 g/dL of lipids, and 0.9 to 1.2 g/dL of protein [1]. Subsequently, breast milk protein is classified into three categories: casein, whey, and mucin, which are present in the milk fat globule membrane. With hundreds of proteins, breast milk offers diverse benefits to infants in the short- and long-term [3]. Protein serves as a source of amino acids, improves the bioavailability of micronutrients, provides immunological protection, stimulates intestinal growth and maturation, forms the microbiome, and improves cognitive abilities [3,4]. A deficiency of protein could inhibit the growth and development of infants and young children [5]. A low-protein diet is strongly associated with stunting in children [5,6]. Protein intake, amino acid availability, and the mechanistic target of the rapamycin complex 1 (mTORC1) pathway play important roles in child growth. When the level of amino acids is low, mTORC1 is spread throughout the cytosol and is inactive. Adequate amino acids translocate mTORC1 to the lysosomal membrane and activate signals in the skeletal muscle [5], which is highly plastic tissue that grows or increases and decreases in size and structure regularly throughout life [7].

Indonesia is a lower-middle income country [8] in which exclusive breastfeeding has reached 74.5%, making breastmilk the primary source of nutrition for infants in the first six months to provide benefits such as immunity with many long-term positive effects such as allergy prevention, lung strengthening, cognitive development, and support for rapid growth. Breast milk provides energy, protein, antibodies, and micronutrients such as vitamins A and E [9]. However, the prevalence of stunting in Indonesia remains relatively high, with a national rate of 30.8% in 2018 and a higher rate of 32.81% in the East Java [10]. There are various causes of stunting, including inadequate intake of nutrients, especially carbohydrates, and protein, apart from infectious diseases [11]. Considering this condition, examining the adequacy of breast milk protein in breastfeeding mothers is necessary.

Protein contents in breast milk are dynamic during lactation and linked to the mother’s diet and the environment [12]. During lactation, the mother needs adequate nutrition for breast development, breast milk volume, and the quality of breast milk [13]. In addition, the supply of nutrients can also regulate the phosphorylation state of proteins involved in the activation of protein synthesis [14]. Breastfeeding mothers need a protein intake of 65–71 g daily or an additional 17 g per day [15]. Amino acids, especially essential amino acids, play an important role in the synthesis of milk protein and are able to increase the production of milk and milk protein [16]. Moreover, consuming high-protein foods can increase the prolactin levels in the body. Prolactin plays a significant role in supplying amino acids through sodium-coupled neutral amino acid transporter 2 (SNAT2) during the protein synthesis process in the mammary glands [14].

The quality of breast milk can be affected not only by the mother’s diet but also by the environment. Although environmental policies have reduced blood lead levels (BLLs) in the general population, lead remains a significant public health concern in developing countries [17]. It is a highly toxic metal that contaminates air, water, and soil [18], and Indonesia has air lead levels ranging from 0.2–2664.2 ng/m^3^, with Surabaya having the highest level compared to other regions [19]. During pregnancy and lactation, lead stored in the bones can be released into circulation if the mother lacks calcium [20]. The toxic effects of lead on the fetus, and the adverse relationships of multiple organs and impairments in neurobehavioural functions have a wide range of exposure without an apparent threshold [21]. However, the Centers for Disease Control and Prevention (CDC) prohibits breastfeeding mothers with BLLs > 40 µg/dL from breastfeeding their babies [22].

Lead can replace calcium through a second messenger mechanism. It can enter cells through calcium channels, bind to calmodulin, and disrupt intracellular calcium homeostasis. As a result of impaired calcium homeostasis, mitochondrial Ca^2+^ increases mitochondrial electron transport and reactive oxidative stress (ROS) generation [23,24]. Oxidants such as ROS can damage almost all cellular components and affect critical biological processes, including protein synthesis [25]. Furthermore, lead contributes to the destruction of existing molecules, such as enzymes, proteins, and DNA [26]. Hydrolyzed proteins and peptides can act as antioxidant molecules by inhibiting lipid oxidation in foods or biological systems through direct radical scavenging [27]. Lead is also an endocrine disruptor [28]. The prolactin levels were significantly lower in the chronic lead-exposed group [29,30], and it is important for glucocorticoid stimulation of milk protein genes in breast epithelial cells [31]. A previous study reported a weak correlation between low lead exposure (1.22 μg/L) and cow’s milk protein content [32]. To date, published studies have identified several factors that influence the protein content of breast milk. However, none of these studies have investigated the correlation between these factors and lead exposure. Moreover, it remains unclear how much protein intake by breastfeeding mothers who are currently exposed to lead is required to maintain breast milk protein levels. Therefore, this study aimed to analyze the correlation between lead exposure and the mother’s protein intake and total breast milk protein and how maternal protein intake could maintain total milk protein levels with increasing maternal BLLs.

## 2. Materials and Methods

### 2.1. Study Participants

This cross-sectional study was conducted from October 2021 to February 2022 at several health centers on the coast of Surabaya, Indonesia. The samples were exclusively breastfeeding mothers who fulfilled the inclusion criteria as follows: (1) having a newborn between 1–6 months; (2) being free from any breast diseases; (3) not consuming a dopamine antagonist; and (4) born at term and breastfed at least eight times per day. The exclusion criteria were not present in all data collection and mothers with restrictive diets. This study involved 110 participants who were selected using a stratified random sampling technique based on their socioeconomic status. Furthermore, Kuppuswamy’s socioeconomic scale 2020 was used to determine the socioeconomic status of the participants [33]. There are two groups of socioeconomic statuses, including low and middle groups, and both groups had the same risk of lead exposure. The exact population number was unknown and therefore the sample size was calculated using the following equation [18]:

$$n=\frac{z^{2}\times p\times q}{d^{2}}$$

where *z* represents the 95% confidence level of 1.96, *p* is the estimated population proportion, *q* is 1 − *p*, *d* represents the limit error (5%), and *n* is the sample size. The proportion of exclusively breastfeeding mothers in the study area was 7.6% one year earlier. According to the calculation, 108 participants were selected. To anticipate sample loss, 20% was added, but 14 people did not meet the inclusion criteria, and 7 people withdrew before the study was completed. Informed consent was obtained from all participants before the study. The Health Research Ethics Commission, Faculty of Medicine, Universitas Brawijaya, authorized this study protocol in number 365/EC/KEPK/12/2021.

### 2.2. Assessment of Maternal Diet

Breastfeeding mothers who agreed to participate completed a sociodemographic questionnaire. They reported their age, education, occupation, child number or parity, and seafood consumption, parity was classified into primiparous (one child) and multiparous (more than one child).

The nutritional evaluation of the maternal diet was determined with multiple 24-h recalls in a week. Three dietary assessments were carried out, one on weekends and two on weekdays. Throughout the interview, breastfeeding mothers were asked about food and drinks consumed a week before BM collection by trained nutritionists. A detailed list of all the foods consumed by the mothers within 24 h was collected. Information was collected about the time consumption, the name of the meals, food portions, food preparation methods, ingredients, and the brand of commercial products. Household measurements and food pictures were used during the interview. The protein, carbohydrate, fat, and calories were calculated using NutriSurvey software and based on the “Indonesian Food Composition” table published by the Indonesian Ministry of Health in 2017 [34]. The calculation of essential amino acids (EAAs) intake was carried out based on “Amino-Acid Content of Foods and Biological Data on Proteins” by the Food Policy and Food Science Service, Food and Agriculture Organization (FAO) of The United Nations [35]. The mother’s current body mass index (BMI) was calculated as the last day of diet assessment weight in kilograms divided by height in meters squared. BMI was classified as underweight (<18.5), normal body weight (18.5 to 24.9), overweight (25 to 29.9), and obese (≥30) [12].

### 2.3. Blood Sample Collection and Measurement

Blood samples were collected concurrently with breast milk samples on the first day after the completion of the morning nutritional assessment (between 8:00–11:00 a.m.) [36]. It was collected at 10 mL in labeled tubes containing ethylene diamine tetraacetic acid (EDTA) and was transported on ice to Prodia’s clinical laboratory. Blood samples were immediately stored at −20 °C. BLLs were measured using inductively coupled plasma–mass spectrometry (ICP–MS) with Agilent 7700 according to the standard protocol for quality control and assurance. The detection limit was 0.09 ng/L, and Prodia was accredited by the College of American Pathologists (CAP) and is ISO 15189 certified, and high-risk blood lead levels were identified at ≥5.0 μg/dL [22].

### 2.4. Breast Milk Sample Collection and Measurement

The breast milk samples were collected (~10 mL) with sterile breast pumping by the trained woman investigators. The samples were collected after the respondent had breakfast and a gap of 30 min after breastfeeding. Breast milk was expressed from the breast that was not recently breastfed, on the day after the mother’s diet was measured. The samples were placed in sterile labeled plastic, transported in a cooler bag, maintained at low temperatures, and stored at −80 °C until use. Total protein levels in breast milk were measured using the Bradford method [37], and protein was read with a UV–vis spectrophotometer (Genesys 150, Thermo Fisher Scientific Inc., Vantaa, Finland). The wavelength (λ) used was 595 nm. The breast milk protein was measured in collaboration using the Biochemical Laboratory, Faculty of Mathematics and Natural Sciences, Universitas Brawijaya, ISO 21001 certified.

### 2.5. Statistical Analysis

Data were expressed as the mean and standard deviation (SD) for normal data and expressed as the median and interquartile range (IQR) for abnormal data. The normality of data was assessed using the Kolmogorov–Smirnov test. BLLs were categorized into <2.0 µg/dL, 2.0 to <5.0 µg/dL, and ≥5.0 µg/dL [18]. A Kruskal–Wallis test was used to compare blood lead levels in some participants’ characteristics and to compare the total milk protein in three groups of lead exposure. Furthermore, bivariate correlations between BLLs, maternal diet, and total milk protein were investigated using Spearman correlations. The multivariate linear regression model was used to assess the correlation of all variables of maternal nutrition to total milk protein simultaneously, and whether there was an independent association between maternal protein intake and total milk protein in every group lead exposure after adjustment for important covariates, including maternal age, education, occupation, parity, and current BMI. Subsequently, IBM SPSS statistical software for Windows version 26 was used to conduct the analyses, with the level of statistical significance set at *p* < 0.05.

## 3. Results

### 3.1. Characteristics of Participants

Out of a total of 110 exclusive breastfeeding mothers, 105 (83.63%) were in the age range 20–35 years, 74 (67.27%) were multiparous, 79 (71.82%) had primary and secondary educational levels, and 72 (65.45%) were unemployed (unpublished data). The median age of breastfeeding women was 28 (interquartile range [IQR], 25–33) years, the median number of children was 2 (1–3), and the median current body mass index (BMI) was 24.23 (21.77, 26.80). The median maternal BLLs and total milk protein were 3.3 (2.6–4.6) µg/dL and 1.07 (1.0–1.2) g/dL, respectively (Table 1).

### 3.2. Correlation between Maternal Macronutrient Intake and Total Milk Protein

**Figure 1:**
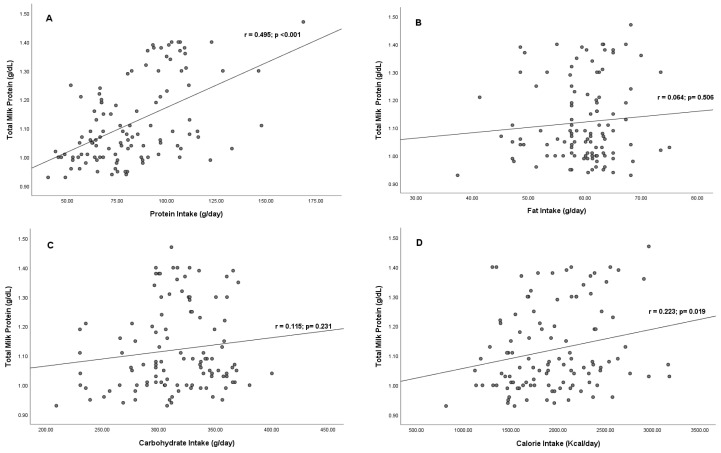
Correlation between maternal diet and total milk protein. (**A**) Scatter plots between protein intake and total milk protein; (**B**) scatter plots between fat intake and total milk protein; (**C**) scatter plots between carbohydrate intake and total milk protein; and (**D**) scatter plots between calorie intake and total milk protein.

The food assessment revealed that 86 (78.18%) respondents ate three times per day with moderate and large portions, and 16 (14.54%) ate more than three times per day. A total of 64 (58.18%) respondents consumed seafood 2–3 times/week, and 23 (20.91%) consumed seafood more than three times every week. None of the respondents consumed calcium supplements or milk specifically for nursing mothers. The median (IQR) protein, fat, carbohydrate intake, and calories were 80.08 (IQR: 64.85–99.96), 60.79 (IQR: 57.56–63.28), 316.30 (IQR: 297.40–343.27) g/day, and 1945.88 (475.77) Kcal, respectively (unpublished data). The Spearman correlation test (Figure 1) showed that protein intake (r = 0.495; *p* < 0.001) and calorie intake (r = 0.223; *p* = 0.019) had a significant positive correlation with total milk protein levels in mature BM. However, fat (r = 0.064; *p* = 0.506) and carbohydrate (r = 0.115; *p* = 0.231) intake did not correlate significantly with total milk protein. The multiple linear regression test showed that maternal nutritional intake increased total milk protein by 33%, and the factor that most affected total milk protein was protein intake (r = 0.554, *p* < 0.001) compared with other variables (Table 2).

According to Table 3, the intake of animal protein (7.88 g/day) was found to be higher than that of plant protein intake. Notably, both animal protein (r = 0.593) and plant protein (r = 0.239) demonstrated significant correlations with total milk protein. Grains, soy, and sea products were identified as the primary sources of protein-rich foods, whereas meat was found to be the least significant source of protein in this study. Spearman’s analysis showed that six essential amino acids from the mother’s diet exhibited correlations with total milk protein. These amino acids included isoleucine (r = 0.331), leucine (r = 0.207), lysine (r = 0.281), phenylalanine (r = 0.245), methionine (r = 0.321), and valine (r = 0.225). On the other hand, there were no significant correlations found between threonine (r = 0.133) and tryptophan (r = 0.185) with total milk protein.

### 3.3. Correlation between Maternal Blood Lead Levels and Total Milk Protein

**Figure 2:**
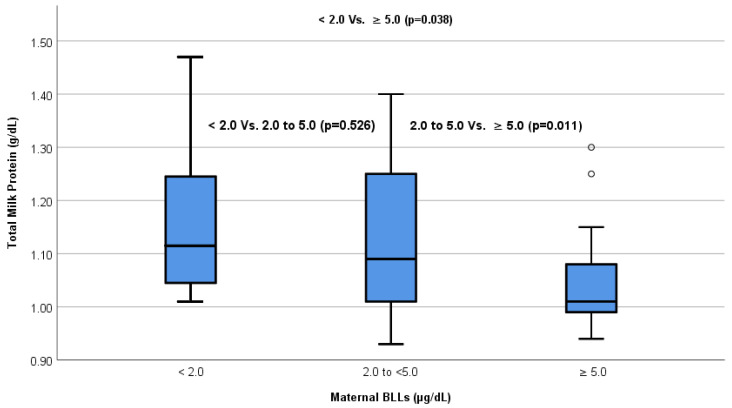
Boxplot of estimated differences in total milk protein in three groups of lead exposure by Dunn’s post hoc test. There was a decrease in median total milk protein in the group with higher lead exposure (<2.0 μg/dL = 1.12 g/dL, 2.0 to <5.0 μg/dL = 1.09 g/dL, and ≥5.0 μg/dL = 1.01 g/dL).

In this study, all respondents were exposed to lead. The median maternal BLLs was 3.3 (interquartile range [IQR], 2.6–4.6) µg/dL. Eight (7.2%) respondents had BLLs <2.0 μg/dL, 81 (73.64%) had BLLs of 2.0 to <5.0 μg/dL, and 21 (19.09%) had BLLs ≥5.0 μg/dL (unpublished data). In this study, maternal BLLs correlated with occupational and seafood consumption. BLLs were higher in employed mothers than in unemployed mothers; 80.95% of working breastfeeding mothers had BLLs of at least 5.0 µg/dL (r = 0.198, *p* = 0.038). In addition, higher seafood consumption correlated with BLLs (r = 0.649, *p* < 0.001). The results of the Spearman correlation test also showed a significant negative correlation between BLLs and total milk protein (r = −0.209; *p* = 0.029). The different Kruskal–Wallis tests showed a significant difference in total milk protein in the groups with lead levels <2.0, 2.0 to <5.0, and ≥5.0 μg/dL (*p* = 0.024) (Figure 2). Dunn’s post hoc test showed that BLLs > 5.0 μg/dL were most influential in reducing total milk protein (*p* = 0.032).

### 3.4. Correlation between Maternal Protein Intake and Total Milk Protein in Each Lead Exposure Group

This study showed that the daily protein intake of mothers could maintain the total protein levels of breast milk in breastfeeding mothers exposed to lead. Meanwhile, in maternal BLLs, the mothers who consumed high daily protein had higher levels of total milk protein than those who consumed low protein. However, at BLLs ≥ 5.0 µg/dL, high protein intake could not maintain breast milk protein levels, and breast milk protein levels decreased. Maternal protein intake had a positive significant correlation with total milk protein in two groups of breastfeeding mothers with lead exposure < 2 μg/dL (*p* = 0.018) and exposure of 2.0 to <5.0 μg/dL (*p* < 0.001) (Table 4).

## 4. Discussion

Heavy metals, such as arsenic (As), mercury (Hg), lead (Pb), and cadmium (Cd), are considered nonbiodegradable pollutants that persist in the environment and pose irreversible toxic effects even at low concentrations. Animal life can be severely affected by their presence [38]. This study showed that all lactating mothers living around Surabaya Beach had been exposed to lead, with a median BLLs 3.3 (IQR, 2.6–4.6) μg/dL. Food is identified as one of the primary sources of lead exposure, with contaminated seafood being the main contributor. The highest seafood lead content is found on the coast of Surabaya, *Anadara granosa* cockle, estimated to have a weekly consumption intake of lead of 8.74 µg/kg body weight [39]. It is uncertain whether the lead exposure results from water pollution, as water lead levels were not measured. The findings indicated that grains, soy, and sea products were the primary source of protein-rich foods in the mother’s diet, while meat was identified as the least significant source of protein. Tempeh and tofu are foods in Indonesia that are rich in protein; the participants consumed this food daily because it is readily available and relatively inexpensive compared to other sources of protein. Tempe is made from soybeans, which contain high isoflavones [40], which are antioxidants that play a role in activating the nuclear E2-related factor 2 (Nrf2)–antioxidant response element (ARE) signaling pathway to modulate the expression of cytoprotective enzymes and counteract oxidative damage [41]. A previous study reported that children consuming more dairy, milk, and yogurt had lower BLLs [36]. Milk, meat, eggs, and soybeans are good sources of food protein to trigger mononuclear phagocyte cell activity to reduce oxidative stress [42]. The CDC promotes the introduction of meat porridge as a nutritional intervention for children with elevated BLLs. Lead can be excreted by the body using chelating agents, and a good chelating agent is easily excreted from the body without further interaction with vital organs and can enter cell membranes to remove intracellular toxic metals. A relatively higher affinity for toxic metals, high lipophilicity, and low molecular size are preferred because they can easily cross the blood-brain barrier. These agents can be given orally, intravenously, or intramuscularly [38].

Exposure to lead has been found to reduce the total protein content of breast milk. This is in line with a previous study by Zhou et al. [32], who provided strong evidence that maternal lead exposure reduces milk protein in animals. The lead mobilization from the bone in the postnatal period is more significant than in pregnancy. The increased release of lead into the blood during the postnatal period is due to increased mobilization of deposited lead into circulation from maternal skeletal and increased bone resorption, leading to an increased risk of miscarriage, low birth weight, and transfer of lead into breast milk. This may be related to inadequate dietary calcium intake [43,44]. Intake of 1200 mg of calcium per day during pregnancy and lactation reduces lead resorption from the mother’s bones. In this study, maternal calcium intake was an average of 940 mg/day, and therefore it is less to fulfill the requirement of the mother’s calcium. Some previous studies have shown that lead exposure could promote apoptosis and inflammation in mouse mammary tissue (in vivo) and cow mammary epithelial cells (in vitro) [45]. Furthermore, lead impacts the synthesis of prolactin, one of the essential hormones in breast milk protein synthesis in breast cells. There is a significant negative relationship between BLLs and serum prolactin levels during pregnancy [30]. Effective interventions to reduce the adverse health consequences of exposure to low-grade lead are still lacking [18]. A recent study reported that the increased protein intake of mothers can maintain milk protein levels exposed to lead, while the exact mechanism requires further exploration.

Human milk protein consists mostly of casein and whey protein, as well as enzymes, endogenous peptides, and mucus derived from the membranes of the milk fat globules. Breast milk protein during lactation functions to fulfill nutrition and has an immunomodulatory effect against pathogens in infants [3]. The composition of breast milk is influenced by other maternal factors, such as diet, age, metabolic health, type of delivery, smoking, stress, and physical activity, and is influenced by physiological factors (lactation or breastfeeding stage), as well as factors related to the infant (such as sex and birth weight) [46]. At present, there is insufficient scientific research available examining the relationship between a mother’s protein intake and total milk protein. This study showed that the maternal diet, such as protein intake, affects breast milk protein levels. However, the intake of fats, carbohydrates, and calories is not correlated with the total protein of breast milk in multivariate analysis. The intake of animal protein exceeded that of plant protein and displayed a significant correlation with the total protein content in breast milk. Animal protein is acknowledged for its superior nutritional value in comparison to plant protein, attributed to factors such as amino acid composition, digestibility, and its role in facilitating the transportation of essential nutrients like calcium and iron [47]. Conversely, vegetable protein tends to have lower concentrations of essential amino acids, particularly methionine, lysine, and leucine, when compared to animal protein [48].

A mother’s diet can affect milk protein through several integrated metabolic pathways [49]. Furthermore, the mother’s food intake in the form of amino acids plays an important role in the milk protein synthesis in the mammary glands. Protein function depends on its amino acid composition [50]. There are nine essential amino acids (EAAs) that the human body cannot synthesize and must obtain from dietary sources. These essential amino acids include histidine, isoleucine, leucine, lysine, methionine, phenylalanine, threonine, tryptophan, and valine. Insufficient intake of EAAs intake can lead to metabolic disturbances that can negatively impact the growth of children [11]. In this study, six essential amino acids from the mother’s diet correlated with total milk protein. These amino acids are isoleucine (Ile), leucine (Leu) [51], lysine (Lys), phenylalanine (Phe), methionine (Met), and valine (Val). However, threonine (Thr) and tryptophan (Trp) showed no correlation with total milk protein. It was observed that phenylalanine levels were high in lactating mothers who consumed significant amounts of tempeh and tofu. These findings align with a previous study by Ding et al. (2010), which reported elevated phenylalanine levels in individuals who consumed soy-based products [52].

The addition of valine (Val), histidine (His), arginine (Arg), tryptophan (Trp), methionine (Met), threonine (Thr), and phenylalanine (Phe) as supplements has been shown to enhance both milk production and milk protein levels [16]. Ile, Leu, Lys, Val, and His have the ability to enhance the transcription of β-casein synthesis by activating the mammalian target of rapamycin (mTOR) and the Ras/ERK signaling pathways, while the Arg signaling pathways can stimulate cell proliferation and enhance protein synthesis by activating the mTOR signaling pathway [16,53,54]. Additionally, Methionine serves as a precursor in the biosynthesis of nitric oxide, which enhances blood flow in the mammary glands [55]. The consumption of high-protein foods can elevate prolactin concentrations, and prolactin acts as a provider of amino acids through SNAT2 during the protein synthesis process in the mammary glands [14]. One of the proteins that has an important role is leucine; leucine plays a significant role in regulating the expression of SNAT2 in the mammary glands, as well as in the liver and adipose tissue, during lactation [51]. Meanwhile, King et al. (2021) found that methionine supplementation may decrease milk protein synthesis but can enhance the availability of other amino acids to fulfill the body’s needs [55]. Furthermore, methionine exhibits antioxidant properties that can mitigate the adverse effects of heavy metal exposure. The combination of methionine and bentonite in a biopolymer nanocomposite has shown the ability to decrease lead ions by as much as 98% [56].

Studies from Europe and the United States found no association between breast milk total protein and a carbohydrate-rich, low-fat diet versus a carbohydrate-rich, high-fat diet [57]. Meanwhile, breastfeeding mothers need a protein intake of 65–71 g daily or an additional 17 g per day [15]. The average protein intake was more than needed, 83.05 ± 23.91 g/day, which supports the breast milk protein level. Subsequently, protein intake positively correlated with total protein in mature breast milk (*p* < 0.001, r = 0,474). Breast milk protein is influenced by the energy content derived from food and the availability of amino acids [58]. A crossover study in Sweden reported that the total protein content of breast milk was higher in mothers on a high-protein diet (8.83 g/dL) than in mothers on a low-protein diet (7.31 g/dL) [49]. Lower protein intake correlated with lower prolactin production, which is needed for protein synthesis in breast epithelial cells [38].

Body mass index is commonly used for evaluating the nutritional status and correlates with measurements of body composition [59]. There was no significant association between maternal BMI and total milk protein [60]. However, this study showed that current BMI was correlated with total milk protein (*p* = 0.010, r = 0.216). This study, in contrast to the findings of Bzikowska et al. [10], revealed that maternal BMI exhibited a stronger positive correlation with milk fat, energy, and dry matter content in human milk than other dietary intakes. Milk protein concentration was found to be higher during the 0–1 week lactation period compared to later periods, while no significant difference was observed for lipids [61]. The longitudinal study showed a strong relationship (*p* < 0.001) between maternal BMI and % fat mass. There was a relationship between higher maternal adiposity and higher concentrations of human milk leptin and protein [62], and fat mass affected protein synthesis. Obesity is associated with amino acid metabolism disorder. Amino acid metabolism-related disorders have been linked to obesity, with elevated amino acid concentrations in both the blood circulation and human milk [63]. Disrupted secretory activation and early cessation of breastfeeding have been attributed to overweight and obesity, potentially due to prolactin resistance, decreased activation of signal transducer and activator of transcription 5 (STAT5), and reduced insulin sensitivity, which can affect breastmilk protein [64]. Human milk from mothers with overweight and obesity has a greater protein content compared with mothers with a normal BMI or underweight [65]. Inconsistencies across studies may be due to different measures of BMI (pregnancy, early postpartum, and current BMI) and sample sizes.

The strengths of this study were that blood and breastmilk samples were collected one day after the mother’s diet assessment was completed, and dietary measurements were carried out three times with a large number of samples. Measuring BLLs using ICP–MS proved to be more sensitive to detecting blood lead levels up to parts per trillion than other tools. However, it is important to note that a cross-sectional study design does not allow causal conclusions. Future research on other heavy metals and breast milk protein is needed to provide a more comprehensive understanding.

## 5. Conclusions

All breastfeeding mothers residing in coastal areas of Surabaya were exposed to lead, which has been shown to reduce total milk protein, specifically in mothers with low protein intake. To anticipate this, high protein intake in breastfeeding mothers is needed to offset the effects of lead exposure. This study highlights the importance of monitoring BLLs in breastfeeding mothers as a precautionary measure in affected areas.

## Figures and Tables

**Table 1:** Characteristics of 110 breastfeeding mothers included in the study.

Characteristic	Frequency	%	Maternal BLLs (Mean ± SD)	*p*-Value ***
Maternal age (year)				
<20	2	1.82	3.75 ± 1.48	0.811
20–35	92	83.63	3.61 ± 1.41	
>35	16	14.55	3.82 ± 1.28	
Parity				
Primiparous	36	32.73	3.48 ± 1.40	0.402
Multiparous	74	67.27	3.73 ± 1.37	
Education level				
Primary *	22	20	4.21 ± 1.32	0.083
Secondary **	57	51.82	3.52 ± 1.41	
University	31	28.18	3.48 ± 1.28	
Maternal Occupation				
Employed	38	34.55	3.45 ± 1.37	0.366
Unemployed	72	65.45	3.73 ± 1.38	
Current BMI (kg/m^2^)				
Underweight (<18.5)	5	4.54	4.02 ± 1.71	0.904
Normal (18.5–24.9)	58	52.73	3.63 ± 1.45	
Overweight (25.0–29.9)	37	33.64	3.58 ± 1.24	
Obese (≥30)	10	9.09	3.80 ± 1.51	
Seafood Consumption (times/week)				
Never-1	23	20.91	2.92 ± 1.07	<0.001
2–3	64	58.18	3.53 ± 1.32	
>3	23	20.91	4.79 ± 1.19	

Note: * Primary: 1–9 years of formal education; ** Secondary: 10–12 years of formal education; BMI: body mass index (calculated as current weight in kilograms divided by height in meters squared, *** Mann–Whitney test for two groups and Kruskal–Wallis test for more than two groups.

**Table 2:** Multivariate analysis of maternal diet and total milk protein.

Variables	Coefficients Correlation	T	*p*-Value	R^2^
Protein Intake	0.554	5.796	<0.001	0.330
Fat Intake	0.051	0.600	0.550	
Carbohydrate Intake	0.076	0.918	0.361	
Calorie intake	−0.128	−1.285	0.202	
Current BMI	0.216	2.619	0.010	

**Table 3:** Food source of protein and amino acids composition of maternal diet.

	Mean ± SD	*p*-Value *
Animal protein (g/day)	45.47 ± 20.10	<0.001
Plant protein (g/day)	37.59 ± 16.45	0.012
Food source of protein (g/day)		-
Fish and sea products	168 ± 14.53	
Eggs	120 ± 17.21	
Meat	23 ± 5.73	
Chicken	80 ± 18.34	
Soy (tempeh, tofu)	180 ± 20.33	
Grains	580 ± 23.17	
Essential amino acid (g/day)		
Isoleucine	3.26 ± 0.93	<0.001
Leucine	5.57 ± 1.99	0.03
Lysine	5.22 ± 1.58	0.003
Phenilalanine	4.03 ± 1.21	0.01
Methionine	1.45 ± 0.47	0.001
Threonine	2.99 ± 0.88	0.167
Tryptopan	1.14 ± 0.69	0.053
Valine	3.13 ± 0.91	0.018

Note: * Spearman correlation with total milk protein.

**Table 4:** Role of maternal protein intake in maintaining total milk protein in some lead exposure groups.

Maternal BLLs (µg/dL)	Maternal Protein Intake	Frequency	Total Milk Protein (g/dL)		
			Mean (SD)	β (SE)	*p*-Value
<2.0	Low	3	1.09 (0.09)	0.943 (0.001)	0.018
	Adequate	1	1.16 (NA)		
	Hight	4	1.19 (0.19)		
2.0 to <5.0	Low	24	1.05 (0.10)	0.460 (0.001)	<0.001
	Adequate	12	1.05 (0.07)		
	Hight	45	1.21 (0.15)		
≥5.0	Low	12	1.03 (0.08)	0.474 (0.001)	0.088
	Adequate	3	1.05 (0.06)		
	Hight	6	1.07 (0.13)		

Abbreviations: BLLs, blood lead levels; NA, not applicable; SE, standard error; SD, standard deviation. Low maternal protein intake was defined as protein intake < 70 g/day, adequate 70–80 g/day, and height > 80 g/day. Linear regression models for total milk protein were performed. Each lead exposure group was adjusted for maternal age, parity, educational level, occupational status, and current BMI. The *p* values showed the interaction between maternal protein intake and total milk protein in each group lead exposure.

## Data Availability

The data generated and analyzed during the current study are included in this published article, and the data used to support the findings are available from the corresponding author upon request.
